# The Fibularis (Peroneus) Tertius Muscle in Humans: A Meta-Analysis of Anatomical Studies with Clinical and Evolutionary Implications

**DOI:** 10.1155/2017/6021707

**Published:** 2017-05-17

**Authors:** Kaissar Yammine, Mirela Erić

**Affiliations:** ^1^The Center for Evidence-Based Anatomy, Sport and Orthopedic Research and The Foot & Hand Clinic, Jdeideh Highway, Fouad Yammine Bld., 1st Floor, Beirut, Lebanon; ^2^Department of Anatomy, Faculty of Medicine, University of Novi Sad, Hajduk Veljkova 3, Novi Sad, Serbia

## Abstract

Being considered an exclusive human structure for a long time, fibularis tertius (FT) is believed to have a secondary function of foot dorsiflexion and eversion. This study is an attempt to approach the issue from an anatomical perspective. A systematic literature search identified 35 studies (7601 legs) which met the inclusion criteria. The weighted results of FT presence were as follows: an “adult cadaveric” frequency of 93.2% and a clinical frequency of 80%. The most common FT origin and insertion sites were the distal half of fibula and the base of the 5th metatarsal, respectively. In 95% of cases, an accessory fibular muscle was detected when FT was lacking. We demonstrated that the discrepancy found between the adult cadaveric and clinical frequency values would point out a probable bias in interpreting previous kinesiological results. On an evolutionary level, comparative anatomy demonstrated a very low FT prevalence among monkeys while reaching a frequency of 30% in gorillas, the only non-human apes having an almost exclusive terrestrial locomotion. The consistent prevalence among humans and the presence of similar functional muscles when it is missing would support an essential role of FT during the phylogenetic development of the erect bipedal posture and probably during gait.

## 1. Introduction 

Since its first description, two issues have been debated over the* “fibularis tertius”* muscle (FT) or* “peroneus tertius”* or* “anterior fibularis”*: whether the FT is a separate entity and whether it is exclusively human. It is most likely that the first description of the PT muscle has been reported by Vesalius [1]. This ninth muscle “employed in the motion of the foot” has been rejected by some anatomists who were contemporaries of Vesalius, such as Colombio and Fallopio [2]. They stated that this “nonus proprius per se musculus” of Vesalius is part of the extensor digitorum muscle that it is inserted on the fifth toe and thus has no separate entity. In spite of later accurate and precise descriptions of FT reported by Albinus [3] and Winslow [4], the FT has been repeatedly negated its separate identity and associated with the extensor digitorum longus (EDL) [5]. In fact, it was not until the nineteenth century that the original description of this muscle as a separate entity has been revalidated by some renowned anatomists such as Henle [6] and Hyrtl [7].

For a quite long time, the muscle was thought to be exclusively a human structure [8–11]. This false statement led some to use it as a proof of a net distinction between humans and other animals [9]. Despite the fact that FT has been occasionally described in some anthropoid apes [12–16], the confusion rose when many authors could not find it in many species of mammals including* Strepsirrhini*, New World monkeys [17] and in Rhesus monkeys [18, 19].

**Figure 1:**
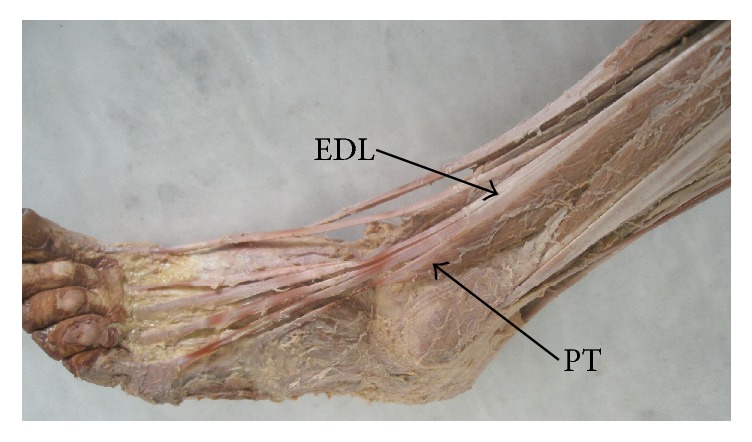
Peroneus tertius and extensor digitorum longus. EDL: extensor digitorum longus; PT: peroneus tertius.

The FT is the most superficial and largest muscle of the anterior group of the leg muscles and it usually arises from the distal third or half of the fibula and of the intermuscular septum [5, 20]. Its tendon runs obliquely and laterally to the most lateral tendon of the EDL (Figure 1). After passing behind the superior and inferior extensor retinaculum, its insertion takes the shape of a broad fan onto the lateral and mediodorsal proximal aspect of the fifth metatarsal and often onto the fascia covering the fourth interosseous space [20, 21]. Its insertion may vary and can go onto the 5th* and* the 4th metatarsal bases or onto the shafts [22]. An additional frequent projection goes towards the base of the fourth metatarsal but could only be visible after lateral mobilization of the FT tendon [5, 23, 24]. Another additional slip to the dorsal aponeurosis of the fifth toe might be sometimes observed [5, 25, 26].

While the function of the FT is eversion of the foot, some researchers showed that subjects without FT are not at higher risk for ankle ligament injury and do not exhibit decreased eversion or dorsiflexion strength [27, 28]. In fact, because of being considered as a secondary gait muscle, studies exploring human gait consistently did not assess the individual action of FT. Nevertheless, FT gained major clinical attention with the advent of ankle arthroscopy, where it constitutes the landmark reference for the anterolateral portal. Additionally, the insertion site of FT is thought by some to be a contributing factor in the case of fractures of the base of the fifth metatarsal bone (Jones fractures) [29]. Its clinical use as a muscle flap to cover soft tissue defect in the foot has been also documented [30, 31].

The cadaveric frequency of FT in adults has been reported to range between 88.2% [23] and 100% [32]. A lower incidence of FT has been found in human fetuses: 78.6% for Sokolowska-Pituchowa et al. [33], 89.7% for Kaneff [34], and 83.16% for Domagala et al. [35]. Its morphology seems to vary largely when comparing study reports.

This study is an attempt to answer a functional question via an anatomical analysis: whether FT has any function in the development of bipedal locomotion and in adult human gait. The aim of this systematic review is to generate more accurate overall, side-based, gender-based, and ancestry-based frequencies of FT along with pooled data on its morphological variants. A comparative anatomical analysis is reported in the discussion; the interpretation of its results along with those of this meta-analysis will try to appraise the functional importance of this muscle.

## 2. Methods

The Checklist for Anatomical Reviews and Meta-Analysis (CARMA) served as the framework for this review [36].

### 2.1. Search Strategy and Identification of Studies

An electronic search strategy was conceived using Medline, Embase, Scielo, EBSCO, and Google Scholar from inception to August 2016. Boolean combinations of the following terms were used: [(“fibularis tertius” OR “peroneus tertius” OR “fibularis anterior”)]. The Digital Collections of the National Library of Medicine, http://www.persée.fr, and http://www.gallica.fr were also searched for old anatomical manuscripts. After deletion of duplicates, title checking was first initiated followed by abstract screening. Abstracts which were found to be likely relevant had their full manuscripts retrieved. Papers reporting at least one primary outcome were included. Reference checking of the included studies was then conducted. No language or age limitations were imposed.

#### 2.1.1. Criteria for Study Selection

All anatomical studies were eligible for inclusion, be it cadaveric, clinical, or radiological, with no restriction to the age of the sample. The primary outcomes were set to be the true or crude prevalence of FT. The true prevalence rate is defined as the number of legs affected compared to the number of legs available for study. The crude FT prevalence is the number of individuals who have either one or two FT compared to the number of individuals available for study. Secondary outcomes were defined as the ancestry-based, laterality-based, gender-based, and side-based frequencies, size of FT, and its origin and insertion variants. 

*Data Extraction and Analysis*. Data extracted included sample size, baseline demographic characteristics of the sample, and prevalence values. Size, origin, and insertion of PT were also extracted when available. Analysis was performed using StatsDirect v2.7.8 (Altrincham, United Kingdom). Proportion meta-analysis was used to (a) calculate the overall true/crude pooled prevalence estimate (PPE), (b) perform subgroup analysis for side-based, gender-based, and ancestry-based PPEs. Odds ratio (OR) meta-analysis was used to establish potential associations with variables such as sex and side. Heterogeneity was investigated using the *I*^2^ statistic; whenever *I*^2^ > 50%, the random-effect estimate was reported. In case of subgroup analysis of five studies or less, the fixed-effect estimate was used regardless of *I*^2^ value. Sensitivity analyses were performed in two instances, when applicable: a subgroup analysis of studies having samples of more than 100 specimens and another two subgroup analyses of studies published before and after the year 1920.

## 3. Results

### 3.1. Search Results

**Figure 2:**
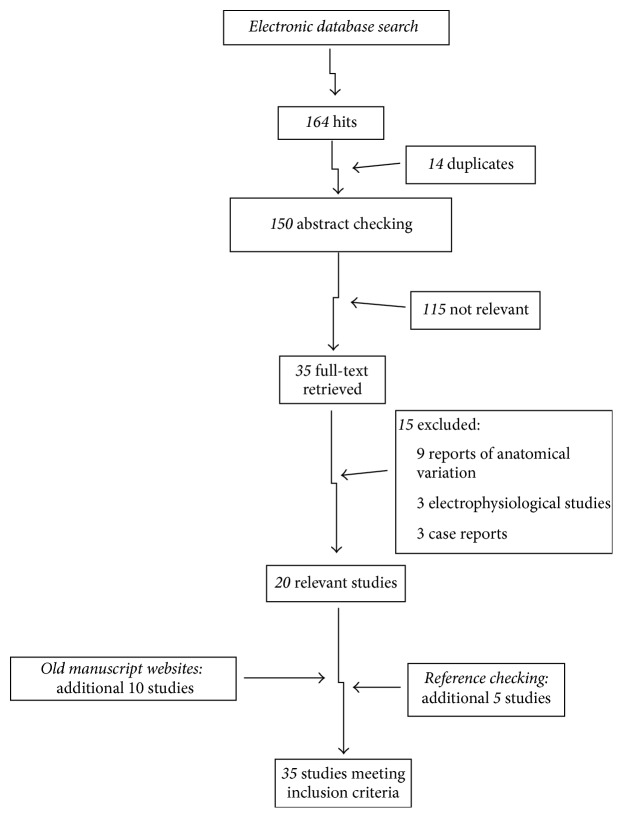
Flowchart of search strategy.

The electronic databases search yielded 164 hits. Fourteen duplicates were removed. Thirty-five studies were considered potentially relevant and twenty met the inclusion criteria. Reasons for exclusion were as follows: 9 reports of anatomical variation, 3 electrophysiological studies, and 3 traumatic clinical case reports. The search of old manuscripts revealed 14 relevant studies where 10 met inclusion criteria. Reference checking of the 30 studies meeting inclusion criteria yielded another 5 studies (Figure 2). In total there were 35 studies: 29 cadaveric (25 adults and 4 embryos/fetuses) and 6 clinical, including 7601 legs.  Table 1 summarizes the characteristics of the included studies.

### 3.2. Prevalence of FT

Table 2 shows the prevalence values of FT reported in the included studies.

#### 3.2.1. Adult Cadaveric Studies

Twenty-five studies [5, 20, 21, 23, 25, 26, 30–32, 37–52] including 3628 legs yielded a weighted true prevalence of 93.2% (95% CI = 0.916 to 0.945, *I*^2^ = 62.2%). A sensitivity analysis of 9 studies including samples of more than 100 leg specimens with a total of 2517 specimens yielded a weighted frequency of 92.7% (95% CI = 0.908 to 0.943, *I*^2^ = 63.2%). Eight studies published before the year 1920 with a total of 1723 legs yielded a weighted frequency of 93.3% (95% CI = 0.920 to 0.944, *I*^2^ = 39.3%). Seventeen studies published after the year 1920 with a total of 1805 legs yielded a weighted frequency of 93.7% (95% CI = 0.915 to 0.956, *I*^2^ = 6.8%).

Eight studies [23, 25, 32, 37, 38, 42, 49, 52] including 395 cadaver specimen yielded a crude prevalence of 93% (95% CI = 0.88 to 0.964, *I*^2^ = 56.8%).

Thirteen studies [20, 21, 23, 25, 26, 32, 37–39, 43, 49, 51, 52] including 875 right legs and 869 left legs yielded a pooled OR of 1.2 (95% CI = 0.573 to 2.514, *I*^2^ = 68.3, *p* = 0.6). No significance was found in relation to the side.

Nine studies [20, 23, 25, 37–39, 43, 49, 52] including 622 males and 334 females yielded a pooled OR of 1.77 (95% CI = 1.035 to 2.691, *I*^2^ = 0%, and *p* = 0.03) in favor of male sex.

Thirteen “Caucasian” studies [5, 20, 23, 25, 30, 37–39, 44–47, 49] including 1788 legs yielded a true frequency of 92.3% (95% CI = 0.910 to 0.934; *I*^2^ = 0%).

Four Indian studies [26, 50–52] including 474 legs yielded a true frequency of 90.8% (95% CI = 0.880 to 0.932, *I*^2^ = 82.4%).

Three South American studies [31, 32, 48] including 188 legs yielded a true frequency of 97.4% (95% CI = 0.916 to 0.998, *I*^2^ = 67.5%).

Two Japanese studies [40, 41] including 938 legs yielded a true frequency of 95.5% (95% CI = 0.940 to 0.9670, *I*^2^ = 36.5%).

One Turkish study [21], one African study [42], and one Chinese study [43] reported true frequencies of 95.4%, 90.2%, and 89.3%, respectively.

#### 3.2.2. Fetal Cadaveric Studies

Four studies [33–35, 53] with a total of 570 legs reported a true prevalence of 82.1% (95% CI = 0.788 to 0.851, *I*^2^ = 0%).

#### 3.2.3. Clinical Studies

Six studies [27, 28, 54–57] with a total of 3160 legs yielded a true frequency of 80% (95% CI = 0.625 to 0.914, *I*^2^ = 98.6%).

All clinical studies but Bourdon and Petitdant [56] with a total of 1537 subjects yielded a crude frequency of 75.7% (95% CI = 0.578 to 0.898, *I*^2^ = 97.5%).

### 3.3. Morphology of FT

Table 3 shows reported origin, insertion, and size of FT from the included studies.

#### 3.3.1. Origin of FT

Eleven studies [26, 30, 31, 34, 35, 47–52] reported the origin of PT. Out of 1026 observed FT, 854 (83.2%) originated from the fibula with 721 (70.2%) originated from the distal half of the fibula and 133 (13%) from the distal third. Additionally, 162 (15.8%) originated from the EDL muscle.

#### 3.3.2. Insertion of FT

All studies but that of Bhatt et al. [50] reported the mode of insertion. Out of 1248 observed FT, in 152 (12.2%) of cases the FT inserted on the shaft of M5, 252 (20.2%) took insertion on the base of M5 (5th metatarsal), 292 (23.4%) on base and shaft of M5, 423 (33.9%) on both M4 (4th metatarsal) and M5, 38 (3%) on M4, and 24 (2%) on EDL tendon. In 67 (5.3%) cases, the distal tendon of FT gives a slip onto the head of M5 or onto the base of the fifth toe.

#### 3.3.3. Size of FT

Six studies [21, 31, 48–50, 52] with a total of 313 FT yielded a mean tendon length of 5.62 cm. Five studies [21, 31, 47, 49, 52] with a total of 279 FT yielded a mean width of 3.28 mm.

#### 3.3.4. Number of FT Tendon Slips

Eleven studies [20, 21, 23, 26, 34, 37, 38, 47, 49, 51, 52] reported the number of FT slips with a total of 795 FT; 699 FT (88%) had a single slip and 91 (12%) a double slip. Out of the 91 double slips, 4 (4.4%) were considered as double tendons.

#### 3.3.5. Number of Accessory Muscles When FT Is Absent

The study of Johnson [20] reported systematically an accessory peroneal tendon when FT was lacking (7 cases); 5 cases displayed a fibularis digiti minimi tendon and 2 cases had an associated fibularis quartus muscle and tendon, all taking insertion onto the base/shaft of the 5th metatarsal. Out of 14 cases [23, 37, 38] with an absent FT, 11 (78.5%) cases demonstrated replacement with a fibularis digiti quinti and 2 (14.3%) cases showed a tendon slip from fibularis brevis (PB). In total, in 20 (95.2%) cases where FT was absent, an accessory peroneal muscle or a tendinous slip from PB to the 5th metatarsal was present.

#### 3.3.6. Tendon Connections from EDL When FT Is Absent or Thin

Joshi et al. [26] reported that in both cases where the FT was absent, it was replaced by a slip from the lateral margin EDL. In two cases where FT was very thin, an intertendinous connection emerging from the lateral tendon of EDL was found joining that of the weak FT.

## 4. Discussion

This meta-analysis demonstrated that FT is highly prevalent in humans (93%). This value varied insignificantly when compared to those of the two subgroup sensitivity analyses: the one including samples of more than 100 specimens and the other comparing it to studies published before 1920. The weighted frequency of 93% is found to be higher than that reported in some reviews and textbooks. For instance, low absence frequencies were reported by Kimura and Takahashi [16] and Williams et al. [58], 4.8% and 4.4%, respectively, whereas Hansen Jr. [59] and Sarrafian [60] stated a prevalence of 90%. Moreover, Wood Jones [61] described its absence in 15% of cases while Romanes [62] reported that the muscle is often absent. This study shows that FT absence is rather the exception. It is worth noting that few old textbooks reported frequencies close to that found in this study [63–65] but these are seldom consulted by students [5]. FT frequency was found to vary between ancestries; South American and Japanese populations had the highest frequency values (97.4% and 95.5%, resp.) while Africans, Indians, and Chinese showed the lowest values (90.2%, 90.8%, and 89.3%, resp.). While no difference was found in relation to the side, a mild association was present in favor of the male sex. Therefore, FT frequency could have a genetic basis which might explain the observed variation between populations. On the other hand, the fetal FT frequency is found to be 11% lesser when compared to that of the adult. Contrary to the findings of Domagala et al. [35] who reported nonsignificant prevalence value differences between age categories of their fetal material, Kaneff [34] rarely found a FT in fetuses less than 40 mm of length (less than 10 weeks of age). The latter finding could explain the difference observed between adult and fetal frequencies. Kaneff [34] also demonstrated that the individualization of FT tendons started at the proximal third of the fifth metatarsal (from the distal slip to the fifth toe of the EDL) and continues distally towards the toes before proceeding proximally for the separation with the EDL belly in the leg at a later stage.

Interestingly, cadaveric dissection revealed a higher frequency (93%) when compared to that obtained with clinical examination (80%). Thus, it seems that around 13% of FT could be missed by routine clinical tests. However, it has been demonstrated that FT tendon and/or FT belly are sometimes difficult to separate from the extensor digitorum longus [30]. That could account for the lower clinical frequency value obtained in this review where palpation might not differentiate FT tendon from the lateral slip of the EDL. Additionally, too many variants of distal FT insertion have been observed with up to nine categories of insertional type [34] where some tendons could be very thin, which might add to the clinical bias. A negative clinical test could have an impact on surgical procedures such as ankle arthroscopy. In fact, the FT is the landmark for the anterolateral portal and the latter is usually used for placement of the inflow cannula. Furthermore, the discrepancy between the cadaveric and clinical frequency values would also indicate a bias when assessing FT function. For instance, results from clinical studies showing no association between absent FT and decrease in foot eversion and dorsiflexion [27, 28] should be reinterpreted in the light of our findings. This negative correlation could be simply due to missed PT tendons on clinical examination.

On the other hand, origin and insertion sites as found in this study were not similar to those reported in many anatomy textbooks. The majority of FT in this study were found to originate from the distal half, rather from the usually reported distal third or fourth of the fibula [58, 66]. More, some textbooks [67–70] did not mention the 4th metatarsal neither the combined proximal parts of the 5th* and* 4th metatarsals as possible sites of insertion.

Interestingly, this study showed that, in more than 95% of cases where FT is missing, an accessory fibular muscle or a tendinous slip from EDL was present. We believe that the findings of Johnson [20] of a constant presence of an accessory fibular muscle whenever FT is absent should incite future authors to investigate the presence of such accessory muscles when studying FT. In fact, Macalister [24, 71] reported the same observations in all the cases where FT muscle was absent. It is worth noting that both variants of accessory fibular muscles often share close origin and insertion sites as FT [72]. We argue that, in cases where FT is absent, these accessory muscles are likely to be considered as compensatory structures. Again, the presence of these accessory muscles would have introduced bias when clinically evaluating ankle/foot range of motion; studies such as reported by Witvrouw et al. [27] and Oyedun et al. [28] did not attempt to locate the presence of an accessory fibular muscle prior to their clinical investigation.

Additionally, many of the included studies described FT insertion as broad and fan-shaped; Krammer et al. [5] compared it to a “pes anserinus.” However, others just reported its main distal attachment with no further details. Those studies which provided detailed descriptions of FT insertion attachments reported a combined insertion onto the 5th and 4th metatarsal bases in approximately 34% of cases. Krammer et al. [5] were able to find such insertion pattern in all subjects; these authors stated that a simultaneous insertion on the 4th metatarsal becomes visible only after mobilization and lateral shifting of the FT tendon. We argue that such broad insertion would increase the truss over the lateral mid-foot and therefore would increase the eversion action of FT.

On an evolutionary level, FT is not found in* Strepsirrhini *and New World monkeys as stated earlier. Two single instances in Old World monkeys were reported in the pig tailed baboon* (Papio ursinus)* [73] and in the Toque monkey* (Macaca sinica)* [17]. Additionally, Kimura and Takahashi [16] observed FT in five legs (2.9%) of three male specimens (3.4%) out of 174 legs of 87 (45 males and 42 females) Crab-eating monkeys* (Macaca fascicularis)*. In great apes, FT has been found with higher frequencies. Loth [74] reported its presence in 1 out of 15 Orangutans (6.6%) from a single study. This author stated that the muscle has been observed in 5% of* chimpanzees*. Kimura and Takahashi [16] completed the review of Straus Jr. [14] and concluded that FT has a frequency of 29.6% (8 out of 27 dissected specimens) in* gorillas*. It is very likely that the presence of PT is associated with the bipedal type of locomotion. The prevalent presence of this muscle in modern humans and* gorillas*, exclusively and mostly terrestrial, respectively, would point out an evolutionary acquisition related to “bipedalism” [5]. The same rationale could be applied to* chimpanzees* where their lesser presence is likely due to the fact that those apes are not exclusively terrestrial: being both, arboreal* and* terrestrial. In fact, foot inversion due to dorsiflexion is not counteracted by any muscle in arboreal apes [75]. When comparing humans to non-human primates, who are able to manifest some form of bipedal locomotion, Romanes [62] ascribed the eversion function of FT as a characteristic feature of human locomotion. Jungers [76] labeled it as a swing-phase muscle acting to level the sole of the foot prior to the next touchdown in humans; its everting activity counters the inverting action of the tibialis anterior. However, during the swing phase of bipedal walking of non-human primates with no FT, these authors were able to record a higher recruitment of fibularis longus and brevis. The increased foot eversion (clearing the toes of the ground) during the swing phase would maximize the plantar weight baring surface during the stance phase. It has been noted that while elevating and pronating the lateral border of the forefoot, FT action would shift the line of weight balance towards the medial arch of the foot to maintain balance during the stance phase [76, 77]. Thus, FT could help improving the economy of bipedal walking.

The evolutionary, functional, and morphological interpretations made in the light of our results concur to conclude that FT is an important functional anatomical structure. Its high frequency, the presence of other similar muscles when absent, and its broad insertion on the lateral aspect of the mid-foot would confer to this muscle an essential role in human bipedalism. By using quantitative evidence synthesis, the findings of this meta-analysis coupled with FT frequencies found in primates indicate that FT function would have had an essential role during the phylogenetic development of the erect bipedal posture, a statement which is in line with that made by Krammer et al. [5]. These authors also claimed that FT action could be fundamental during postnatal development of the human gait and could highly contribute to the efficiency of adult gait. The fact that subjects lacking FT do not demonstrate gross gait abnormalities would be inconsistent with FT being a fundamental gait muscle. Nevertheless, the findings here indicate that accessory peroneal muscles are quite always present when FT is absent. These muscles could substitute a missing FT function and might mask the lacking action of an absent FT. Additionally, the above search literature strategy could not find reports analyzing individual FT action during gait. Being considered as a secondary extensor and evertor muscle, the published studies systematically included FT with the other peroneal muscles when studying human walking. Lastly, the high prevalence of FT observed in humans and its consistency between different ethnicities do not suggest an ongoing process of phylogenetic degeneration as it is the case of palmaris longus; on the contrary, it could imply a necessary function still to be secured by this muscle.

## 5. Conclusion

The interpretation of the meta-analytical and comparative results would point out the fact that such a recent and constant muscle might have an important function, or a fine-tuning function, related to human motion, be it during walking and/or during faster paces of motion such as running. Due to our “important gait muscle” argument and to the lack of reported individual analysis in human gait, further research is warranted to assess FT contribution during the different types of human locomotion.

## Figures and Tables

**Table 1:** Characteristics of the included studies.

Studies	Population	Study type	Age (y)	Sample size: subjects	Male	Female	Sample size: legs	Right	Left
Adachi, 1909	Japanese	Cadaveric	Adults	—	—	—	630	—	—
Ashaolu et al., 2013	Nigerian	Clinical	16–25	100	47	53	200	100	100
Bertelli and Khoury, 1991	French	Cadaveric	Adults	22	—	—	44	22	22
Bhatt et al., 2010	Indian	Cadaveric	Adults	—	—	—	94	—	—
Bourdon and Petitdant, 2012	French	Clinical	20.43	86	26	60	86	—	—
de Gusmão et al., 2013	Brazilian	Cadaveric	Adults	—	—	—	64	32	32
Domagala et al., 2003	Polish	Cadaveric	Fetuses	33	—	—	66	33	33
Domagala et al., 2006	Polish	Cadaveric	Fetuses	193	96	97	386	193	193
Ercikti et al., 2016	Turkish	Cadaveric	Adults	17 (+11 legs)	—	—	44	23	21
Johnson, 1973	Caucasians (94%)	Cadaveric	Adults	31 (+19 legs)	42	39	81	40	41
Joshi et al., 2006	Indian	Cadaveric	Adults	110	—	—	220	110	110
Kaneff, 1980	French	Cadaveric	Fetuses	—	—	—	34	10	24
Koganei et al., 1903	Japanese	Cadaveric	Adults	—	—	—	308	—	
Krammer et al., 1979	Austrian	Cadaveric	Adults	—	—	—	169	—	—
Larico and Jordan, 2005	Bolivian	Cadaveric	Adults	46	—	—	92	46	46
Le Double, 1897	French	Cadaveric	Adults	120	60	60	240	120	120
Loth, 1913	African	Cadaveric	Adults	56	—	—	112	56	56
Marin et al., 2006	Brazilian	Cadaveric	Adults	16	16	0	32	16	16
Nakano, 1923	Chinese	Cadaveric	Adults	39 (+6 legs)	39	6	84	40	44
Oyedun et al., 2014	Nigerian	Clinical	15–70	169	115	54	338	169	169
Posmykiewicz, 1934	Polish (907)	Clinical	Adults	1000	598	402	2000	1000	1000
	Jews (93)								
Ramirez et al., 2010	Chilean	Clinical	18–26	168	68	100	336	168	168
Reimann, 1981	German	Cadaveric	Adults	—	—	—	200	—	—
Rourke et al., 2007	British	Cadaveric	Adults	41	22	19	82	41	41
Schwalbe and Pfitzner, 1894	German/French	Cadaveric	Adults	—	363	174	537	273	264
Sokolowska-Pituchowa et al., 1974	Polish	Cadaveric	Adults	—	—	—	101		
Sokolowska-Pituchowa et al., 1979	Polish	Cadaveric	Fetuses	42	—	—	84	42	42
Stevens et al., 1993	British	Cadaveric	62–100	—	20	20	40	35	5
Surekha et al., 2015	Indian	Cadaveric	45–70	50	50	0	100	50	50
Verma and Seema, 2015	Indian	Cadaveric	30–70	30	28	2	60	30	30
Werneck, 1957	Caucasian (mainly) and Black	Cadaveric	5 months–62 y	45	—	—	90	45	45
Witvrouw et al., 2006	Belgian	Clinical	17–21	100	50	50	200	100	100
Wood, 1866	British	Cadaveric	Adults	32	28	4	64	32	32
Wood, 1867	British	Cadaveric	Adults	34	22	12	68	34	34
Wood, 1868	British	Cadaveric	Adults	36	18	18	72	36	36

**Table 2:** Prevalence values of PT.

Studies	Population	Sample size: subjects	Crude prevalence	Sample size: legs	True prevalence	Right prevalence	Left prevalence	Male prevalence	Female prevalence
Adachi, 1909	Japanese	—	—	630	598 (95%)	—	—	—	—

Ashaolu et al., 2013	Nigerian	100	73 (73%)	200	125 (63%)	67 (67%)	58 (58%)	62 (66%)	63 (59.4%)

Bertelli and Khoury, 1991	French	22		44	40 (91%)	—	—	—	—

Bhatt et al., 2010	Indian	—	—	94	84 (89.4%)	—	—	—	—

Bourdon and Petitdant, 2012	French	86	—	86	76 (88.4%)	—	—	21 (80.8%)	55 (91.7%)

de Gusmão et al., 2013	Brazilian	—	—	64	62 (96.9%)	—	—	—	—

Domagala et al., 2003	Polish	33	—	66	52 (78%)	—	—	—	—

Domagala et al., 2006	Polish	193	—	386	321 (83.2%)	—	—	—	—

Ercikti et al., 2016	Turkish	17 (+11 legs)	—	44	42 (95.5%)	22 (95.6%)	20 (95.2%)	—	—

Johnson, 1973	Caucasians (94%)	31 (+19 legs)		81	74 (91.3%)	34 (85%)	40 (97.56%)	39 (92.8%)	36 (92.3%)

Joshi et al., 2006	Indian	110	—	220	197 (89.55%)	96 (87.3%)	101 (91.8%)	—	—

Kaneff, 1980	Caucasians	—	—	34	30 (88.2%)	—	—	—	—

Koganei et al., 1903	Japanese	—	—	308	298 (96.7%)	—	—		

Krammer et al., 1979	Austrian	—	—	169	157 (92.9%)	—	—	—	—

Larico and Jordan, 2005	Bolivian	46	46 (100%)	92	92 (100%)	46 (100%)	46 (100%)	—	—

Le Double, 1897	French	120	109 (90.8%)	240	226 (94.2%)	113 (94.2%)	113 (94.2%)	55 (91.7%)	54 (90%)

Loth, 1913	African	56	50 (89.3%)	112	101 (90.2%)	—	—	—	—

Marin et al., 2006	Brazilian	16	—	32	30 (94%)	—	—	—	—

Nakano, 1923	Chinese	39 (+6 legs)	—	84	75 (89.29%)	37 (92.5%)	38 (86.36%)	32 (82.05%)	5 (83.33%)

Oyedun et al., 2014	Nigerian	169	140 (82.8%)	338	299 (88.46%)	148 (87.6%)	151 (89.35%)	102 (88.7%)	49 (90.7%)

Posmykiewicz, 1934	Polish (907) Jews (93)	1000	913 (91.3%)	2000	1852 (92.6%)	925 (92.5%)	927 (92.7%)	1119 (93.56%)	733 (91.17%)

Ramirez et al., 2010	Chilean	168	83 (49.11%)	336	171 (50.89%)	84 (50%)	81 (48%)	—	—

Reimann, 1981	German	—	—	200	180 (90%)	—	—	—	—

Rourke et al., 2007	British	41	38 (92.7%)	82	77 (93.9%)	38 (92.7%)	39 (95.1%)	21 (95.4%)	17 (89.5%)

Schwalbe and Pfitzner, 1894	German	—	—	537	493 (91.8%)	252 (92.3%)	241 (91.3%)	339 (93.4%)	154 (88.5%)

Sokolowska-Pituchowa et al., 1974	Polish	—	—	101	93 (92%)	—	—	—	—

Sokolowska-Pituchowa et al., 1979	Polish	42	—	84	66 (78.6%)	—	—	—	—

Stevens et al., 1993	British	—	—	40	38 (95%)	—	—	—	—

Surekha et al., 2015	Indian	50		100	87 (87%)	45 (51.72%)	42 (48.27%)	—	—

Verma and Seema, 2015	Indian	30	30 (100%)	60	60 (100%)	28 (100%)	2 (100%)	30 (100%)	30 (100%)

Werneck, 1957	Caucasian (mainly) and Black	45	—	90	86 (95.6%)	—	—	—	—

Witvrouw et al., 2006	Belgian	100	76 (76%)	200	163 (81.5%)	81 (81%)	82 (82%)	41 (87.6%)	41 (87.6%)

Wood, 1866	British	32	30 (94%)	64	61 (95.3%)	30 (94%)	31 (97%)	26 (93%)	4 (100%)

Wood, 1867	British	34	29 (85.3%)	68	60 (88.2%)	30 (88.2%)	29 (85.3%)	20 (91%)	9 (75%)

Wood, 1868	British	36	33 (91.7%)	72	69 (96%)	33 (91.7%)	36 (100%)	17 (94.4%)	16 (88.9%)

**Table 3:** Origin, insertion, and size of PT.

Studies	Nb of observed PT	Origin *N* (%)			Insertion *N* (%)					Tendon size	
		Distal half fibula	Distal third fibula	EDL tendon	Shaft M5	Base M5	M4-M5	M4	Tendon of the EDL	Length (cm)	Width (mm)
Bertelli and Khoury, 1991	40	40 (100)	0	0	4 (10)	32 (80)	0	0	4 (10)	NR	NR

Bhatt et al., 2010	84	78 (92.8)	0	6 (7.14)	NR	NR	NR	NR	NR	6.4	NR

de Gusmão et al., 2013	62	28 (45.2)	34 (54.8)	0	48 (77.4)	0	14 (22.6)	0	0	1.2	4.5

Domagala et al., 2006 (fetuses)	321	180 (56)	0	141 (44)	32 (9.9)	0	289 (90)	0	0	0.36–1.7	NR

Ercikti et al., 2016	42	NR	NR	NR	0	R 15 (35.8)	R 3 (7.1)	R 4 (9.5)	0	R 6.22	R 3.1
						L 15 (35.8)	L 4 (9.5)	L 1 (2.4)		L 5.77	L 3.3

Johnson, 1973	74	NR	NR	NR	9 (10.9)	35 (47.3)	10 (12.4)	1 (1.2)	13 (16)	NR	NR

Joshi et al., 2006	197	R 50 (52.1) L 46 (45.5)	R 46 (47.92) L 55 (54.45)	0	R 33 (34.5)	R 44 (45.8)	NR	NR	NR	NR	NR
					L 27 (26.7)	L 55 (54.4)					

Kaneff, 1980	30	23 (75.7)	0	7 (24.3)	4 (14)	23 (75.7)	3 (10%)	0	0	NR	NR

Marin et al., 2006	30	25 (83.3)	5 (16.7)	0	27 (90)	2 (7)	1 (3)	0	0	8.13	NR

Rourke et al., 2007	77	77 (100)	0	0	0	0	77 (100)	0	0	6.96	4.2

Stevens et al., 1993	38	35 (92.11)	NR	NR	28 (82.5)	NR	NR	NR	4 (10)	NR	3.5 ± 1

Surekha et al., 2015	87	80 (91.9)	0	7 (8.04)	0	39 (44.8)	22 (25.3)	21 (24.1)	0	NR	NR

Verma and Seema, 2015	60	59 (98.3)	0	1 (1.66)	60 (100)		0	0	0	6	5

Wood, 1866	61	NR	NR	NR	55 (90.2)		2 (3.28)	4 (6.5)	0	NR	NR

Wood, 1867	60	NR	NR	NR	56 (93.3)			4 (6.7)	0	NR	NR

Wood, 1868	69	NR	NR	NR	61 (88.4)		2 (2.90)	3 (4.3)	3 (4.3)	NR	NR

NR: not reported, R: right, L: left, M: metatarsal, and EDL: extensor digitorum longus.
